# Increasing Dietary Potassium Enhances Urine Production and Reduces Risk of Calcium Oxalate Stone Formation in Senior Cats

**DOI:** 10.3390/ani16111689

**Published:** 2026-05-31

**Authors:** Jean A. Hall, Shiguang Yu, Alyssa R. Toillion, Dennis E. Jewell

**Affiliations:** 1 Department of Biomedical Sciences, Carlson College of Veterinary Medicine, Oregon State University, Corvallis, OR 97333, USA; 2 Pet Food Division, Taste, Texture & Health ISOL, DSM-Firmenich AG, Wurmisweg 576, 4303 Kaiseraugst, Switzerland; shiguang.yu@dsm-firmenich.com; 3 Pet Nutrition Center, Hill’s Pet Nutrition, Topeka, KS 66617, USA; alyssa_toillion@hillspet.com; 4 Department of Grain Science and Industry, Kansas State University, Manhattan, KS 66506, USA; djewell@ksu.edu

**Keywords:** Feline, urine production, urine relative supersaturation, urine specific gravity, urine stone formation, urolithiasis

## Abstract

The objective of these three studies was to determine if dietary potassium supplementation affects urine production or calcium oxalate (CaOx) relative supersaturation (RSS) in adult cats. In the first study, control cats were fed dry food containing 1.08% potassium (as fed), and treatment cats were fed control food supplemented with KCl (1.95% potassium) for 21 days. In study two, cats were fed one of three treatment foods for two weeks in a three-period crossover study design (control food containing 0.84% potassium, and treatment foods containing control food supplemented with KCl at 1.35% or 1.81% potassium). In study three, control and treatment cats were fed similarly to study one, with each experimental period lasting two weeks in a crossover study design. In study one, urine production (*p* = 0.037), urine potassium excretion (*p* = 0.049), and urine chloride excretion (*p* = 0.007) were greater for cats fed test food. In study two, increasing dietary potassium concentration significantly increased water intake and urine production, and decreased USG in a dose–response manner. Urine concentrations of sodium and phosphorus were significantly decreased by urine dilution. In study three, cats consuming test food had a 25% increase in urine production and reduced urine specific gravity compared with cats consuming the control food. In addition, CaOx RSS was reduced (*p* = 0.007). No adverse effects were observed in healthy cats consuming increased dietary potassium.

## Simple Summary

A series of three studies was performed in healthy adult cats to determine if dietary potassium supplementation could safely be used to increase water intake and urine production, and thereby dilute urine mineral concentrations as a means of preventing urinary calculi in cats. The first study was a two-group comparison between control cats and treated cats; the second study was a three-group, dose–response, cross-over study with control cats and treated cats fed with two levels of dietary potassium; and the third study was a cross-over study between control cats and treated cats. We found that up to 1.95% potassium in dry food, as fed (compared to control cats eating a dry food containing 1.08% potassium, as fed), is beneficial for increasing water intake and urine production, for decreasing urine specific gravity and concentrations of urine minerals, and for reducing calcium oxalate relative supersaturation. Supplementing cat food with potassium chloride was safe and should result in less stone formation in cats.

## 1. Introduction

Feline lower urinary tract disorders represent a spectrum of conditions characterized by hematuria, dysuria, pollakiuria, and partial or complete urethral obstruction. In domestic cats, struvite and calcium oxalate stones are the most common uroliths [1,2,3]. Increased water intake and decreased urine concentration are recommended for cats to prevent urolithiasis [4]. Increasing urine volume may dilute urine mineral concentrations and prevent them from reaching their supersaturation concentrations and forming stones.

Dietary prevention and management of urolithiasis includes adjusting urine pH, controlling stone-forming elements, using stone-forming inhibitors, and increasing water intake and urine volume to dilute urine. Researchers have employed several strategies to increase water intake in cats, including increasing food moisture through the use of high-moisture food [5], increasing dietary protein [6], increasing dietary salt intake [7,8], and varying the water source [9,10]. Nutrient-enriched water has also been utilized to improve measures of hydration in healthy cats [11]. Another strategy that we reported on previously involved the use of viscous water to take advantage of the unique anatomy of the cat’s tongue [12]. Increasing the viscosity of water with an ingredient that is palatable to cats increased their water intake by allowing cats to lift more water per lap, thus lowering the calculated risk of urine stone formation [12]. However, some of these strategies are not very effective or have limitations. For example, increasing dietary protein concentration in cats increases urine volume, but also increases urine calcium concentration, renal calcium and oxalate excretion, and urine calcium oxalate (CaOx) relative supersaturation (RSS), all of which favor the development of CaOx uroliths [13].

In one study, an energy-dense dry cat food (4072 kcal/kg food, as fed) designed for control of urolithiasis, and with increased sodium content > 1.2% (3.27 g/Mcal, as fed), was effective at increasing water intake and urine production [14]. In a recent study evaluating the nutritional adequacy of commercial cat foods [15], sodium content ranged from 0.90 to 1.47 g/Mcal of metabolizable energy (mean 1.21 g/Mcal; Association of American Feed Control Officials [AAFCO] minimum: 0.50 g/Mcal). Yet, the potential health risk of salt-supplemented cat food cannot be ignored in older cats prone to chronic kidney disease. In another study, cats with renal insufficiency eating a food high in sodium (1.10%, as fed) showed an increase in azotemia biomarkers and phosphorus concentrations compared with cats eating a lower sodium food (0.35%, as fed) [16]. Substituting potassium for sodium in cat foods is an alternative that could benefit cats relying on dietary management to increase water consumption and urine output. In the same study evaluating the nutritional adequacy of commercial cat foods [15], potassium content ranged from 1.33 to 2.34 g/Mcal of metabolizable energy (mean 1.62 g/Mcal; AAFCO minimum: 1.50 g/Mcal). Therefore, the objective of these studies was to evaluate whether increasing dietary potassium concentrations from 1% to 2% affects urine production in adult cats. We also investigated whether there is a dose–response effect at a moderate increase (1.4% potassium), and determined if dietary potassium supplementation alters CaOx or struvite RSS in healthy adult cat urine.

## 2. Materials and Methods

### 2.1. Animals

All study protocols were reviewed and approved by the Institutional Animal Care and Use Committee, Hill’s Pet Nutrition, Inc., Topeka, KS, USA (Permit Numbers: CP20027, CP28651, CP20798), and complied with the National Institutes of Health Guide for the Care and Use of Laboratory Animals [17]. Cats were individually housed for the majority of the day in spacious indoor rooms with natural light that varied with seasonal changes. All cats were provided with regular opportunities for socialization and environmental enrichment by caretakers, allowing cats to mingle twice a day. Cats experienced behavioral enrichment through interactions with each other, by daily interaction and playtime with caretakers, and by daily opportunities to exercise on enclosed sun porches. Indoor room temperatures were kept constant, although cats would have been subject to more varied temperatures if outside sunning on porches. Cats were owned by the commercial funders of this research or their affiliates, who gave permission for them to be included in this study. At the conclusion of each study, all cats were returned to the Hill’s Pet Nutrition, Inc. (Topeka, KS, USA) colony.

Each cat had an annual physical examination, complete blood count (CBC), serum biochemical analyses, urinalysis, and urine culture if indicated by the urinalysis results. Cats enrolled in a study could subsequently be removed from the study if they experienced a loss of >10% body weight despite intervention, if they stopped eating or ate < 50% of the food ration for 3 consecutive days, if they were diagnosed with any secondary systemic disease, or if an adverse event occurred that required removal from the study at the discretion of the attending veterinarian.

### 2.2. First Study Design

Twelve adult domestic short-hair (DSH) cats were chosen from the colony and randomly divided into two groups of six cats each. Control cats were 13.5 ± 1.3 years of age (mean ± SEM), 4 neutered males and 2 ovariohysterectomized females, with an initial body weight of 4.79 ± 0.41 kg. Test food cats were 11.9 ± 1.2 years of age (*p* = 0.38), 4 neutered males and 2 ovariohysterectomized females, with an initial body weight of 5.11 ± 0.41 kg (*p* = 0.58). The control group of six cats was fed dry food containing 1.08% potassium (as fed), and the test group of cats was fed the control food supplemented with KCl, such that the final concentration was 1.95% potassium (as fed), for 21 days. Control food was a commercial adult cat food designed to help dissolve struvite uroliths (Hill’s Pet Nutrition, Inc., Topeka, KS, USA). The amount of potassium in the food was verified by a commercial laboratory (Woodson-Tenet, Des Moines, IA, USA). The amount of food provided to each cat was adjusted to maintain body weight. Cats had free access to water during the study period. During the last five days of the study period, cats were housed individually so that 24 h collections of feces and urine could be obtained from each cat. Upon observation of a stool, it was scored on a scale ranging from 1 (not solid, >75% liquid) to 5 (cylindrical, >80% firm) as previously described [18].

Food intake was assessed daily, and body weight was assessed weekly. Feces and urine production, and potassium and chloride excretion in feces and urine were assessed during the last five days of the study. Statistical analyses for body weight, food intake, and feces and urine parameters were assessed using a linear mixed model with diet, day, and the interaction as fixed effects in the model. All data are reported as least square means ± SEM. Significance was accepted as *p* ≤ 0.05, whereas *p* ≤ 0.10 was considered a trend.

### 2.3. Second Study Design

Nine adult female DSH cats were chosen from the colony and randomly divided into three groups of three cats each. All but one cat had prior ovariohysterectomy. Cats ranged in age from 10.4 to 18.7 years (Group 1 mean age ± SEM was 15.7 ± 0.69 years; Group 2 was 13.1 ± 1.33 years; Group 3 was 16.2 ± 1.50 years). A 3-treatment, 3-period crossover design was used, whereby each cat was fed one of three treatment foods according to the randomly assigned treatment sequence. Each treatment period was two weeks. The first week was used as a washout period, and the second week as a data-collection period. One group of three cats was fed a dry control food containing 0.84% potassium (as fed), a second group of three cats was fed control food supplemented with KCl containing 1.35% potassium (as fed), and a third group of cats was fed the control food supplemented with KCl containing 1.81% potassium (as fed). Control food was a commercial adult cat food designed to help dissolve struvite uroliths (Hill’s Pet Nutrition, Inc., Topeka, KS, USA). The amount of potassium in the food was verified by a commercial laboratory (Woodson-Tenet, Des Moines, IA, USA). The amount of food provided to each cat was adjusted to maintain body weight. Cats had free access to water during the study. During week two, cats were housed individually so that 24 h collections of urine could be obtained.

Food and water intake were assessed daily, and body weight was assessed weekly. Twenty-four-hour urine collections in thymol preservative were obtained daily during week two of each treatment period to measure urine production, urine specific gravity (USG), and pH. The mineral concentrations in urine and mineral excretions in urine were also assessed daily during the last five days of the study. Blood was collected for a serum chemistry panel on day 14 of each treatment period.

Statistical analyses for body weight, water intake, food intake, serum analytes, and urinalysis parameters were assessed. Data were analyzed using weekly mean by a linear mixed model with Sequence, Treatment, and Period as fixed effects and Subject (Sequence) as a random effect. The NOBOUND option was used to allow for negative variance component estimates. The Kenward-Roger adjustment was used to calculate the denominator degrees of freedom for the F-tests and SE for the means. The three treatments were compared against each other, and the ADJUST=SIMULATE option was used to control the Type 1 error rate. All data are reported as least square means ± SEM. Significance was accepted as *p* ≤ 0.05, whereas *p* ≤ 0.10 was considered a trend.

### 2.4. Third Study Design

Ten adult female DSH cats were randomly divided into two groups with five cats in each group in a 2-treatment, 2-period crossover design. All cats had prior ovariohysterectomy. Cats ranged in age from 8.4 to 15.7 years (Group 1 mean age ± SEM was 14.3 ± 0.38 years; Group 2 was 12.6 ± 1.43 years). Each treatment period was two weeks, and the foods were crossed between the groups at the end of the first two weeks. The first week was used as a washout period, and the second week as a data-collection period. All cats were housed individually so that 24 h urine collections could be obtained during the last two days of each treatment period. Cats were provided water ad libitum during the study period. The amount of food provided to each cat was adjusted such that its body weight was maintained ±10%. The control food was a dry formula containing 1.08% potassium (as fed). Test food was the control food supplemented with KCl, such that the final concentration was 1.95% potassium (as fed). Control food was a commercial adult cat food designed to help dissolve struvite uroliths (Hill’s Pet Nutrition, Inc., Topeka, KS, USA). The amount of potassium in the food was verified by a commercial laboratory (Woodson-Tenet, Des Moines, IA, USA). Blood was collected for a serum chemistry panel on day 14 of each treatment period.

Statistical analyses for body weight, food intake, urine production, urinalysis parameters, urine mineral concentrations, and serum analytes were carried out. Struvite and CaOx RSS of the urine were also assessed. Data were analyzed using a linear mixed model with sequence, diet, and period as fixed effects and subject (sequence) as a random effect. The NOBOUND option was used to allow for negative variance component estimates. The Kenward-Roger adjustment was used to calculate the denominator degrees of freedom for the F-tests and SE for the means. All data are reported as least square means ± SEM. Significance was accepted as *p* ≤ 0.05, whereas *p* ≤ 0.10 was considered a trend.

## 3. Results

### 3.1. First Study

Control food contained 1.08% potassium and 1% soluble chloride. Test food contained 1.95% potassium and 1.82% soluble chloride (both *p* < 0.001) for cats fed test food (Table 1). Food intake tended (*p* = 0.058) to be greater in cats fed test food, although body weight, feces production, fecal potassium, fecal chloride, and fecal scores were similar between control cats and cats fed the test food. Body weight decreased over the 3-week period in both groups of cats, becoming significantly different from baseline values in control cats (*p* = 0.011) by day 8, and in test food cats (*p* = 0.049) by day 21. Urine production (*p* = 0.037), urine potassium excretion (*p* = 0.049), and urine chloride excretion (*p* = 0.007) were all significantly greater for cats fed the test food.

### 3.2. Second Study

Cats fed food supplemented with KCl had significantly increased water intake and urine production and decreased USG (Table 2). Water intake, urine production, and urine pH increased, whereas USG decreased as intake of dietary potassium increased. The urine concentrations of phosphorus and sodium were significantly decreased by urine dilution associated with increased urine production. Daily urine mineral excretion was increased for potassium and chloride at both supplementation concentrations compared with control food, and for calcium at the higher potassium supplementation concentration. There was no effect of dietary potassium content on food intake or change in body weight among the three treatment groups of cats. Blood chemistry measurements were similar among the three treatment groups of cats except for cholesterol, which was decreased after consuming the highest potassium-supplemented food (Table 3).

### 3.3. Third Study

Cats consuming food supplemented with potassium had significantly increased urine production (increased by 25%) and reduced USG (Table 4). Food intake, fecal score, and body weight were not affected by treatment. Blood chemistry measurements were similar between the two groups of cats, and no adverse events were observed (Table 5). Urine pH was not affected by treatment. As expected, urine potassium and chloride concentrations were significantly increased in cats fed the potassium-supplemented food. Urine concentrations of phosphorus, sodium, oxalate, and sulfate were all significantly decreased in cats fed potassium-supplemented food, whereas other urine minerals measured were not affected. Cats fed food supplemented with potassium tended to have reduced struvite RSS (*p* = 0.071), whereas CaOx RSS was significantly reduced (*p* = 0.007).

## 4. Discussion

In the first study, we showed that cats, like other mammals, maintain potassium homeostasis by eliminating excess potassium via the kidney. Increasing potassium in the food from 1.08 to 1.95% significantly increased urine excretion of potassium and chloride, and increased urine production by 71% in adult cats. Higher dietary intake increased urine excretion, whereas fecal excretion was not affected, as most potassium was absorbed. Potassium is commonly supplemented as KCl. Thus, chloride homeostasis was also assessed. An increase in dietary chloride also significantly increased urine chloride excretion, whereas fecal chloride excretion was not affected. Our results agree with those previously reported for adult cats fed foods containing 0.31% basal potassium and supplemented with 0.5, 0.75, and 1.0% potassium from KCl or KHCO_3_ [19].

A moderate increase in dietary potassium (approximately double) significantly increased urine production in cats, similar to what has been observed in dogs [20]. Urine concentrations of calcium, magnesium, and phosphorus were not measured in this first study. Nonetheless, it would be reasonable to speculate that they would be reduced because of the increased urine volume. Thus, moderately increasing dietary potassium might be beneficial in cats with urolithiasis.

In the second study, we showed that KCl supplementation in the food up to 1.81% had no effect on food intake. In addition, increasing dietary potassium from 0.84 to 1.81% significantly increased water intake and urine production, and decreased USG in a dose–response manner. Measured urine concentrations of phosphorus and sodium were significantly decreased by urine dilution associated with increased urine production at both potassium supplementation concentrations, whereas magnesium concentrations in the urine tended to be decreased (*p* = 0.086) at the higher supplementation concentration (1.81% potassium, as fed). Daily urine mineral excretion was increased for potassium and chloride at both supplementation concentrations compared with control food, and for calcium at the higher potassium supplementation concentration. Others have also noted that renal calcium excretion was higher in cats fed KCl-supplemented foods compared with KHCO_3_-supplemented foods [19]. Urine RSS for CaOx and struvite were not measured in the second study. Again, it would be reasonable to speculate that they would be reduced because of the increased urine volume.

In the third study, the effect of KCl-supplemented food on CaOx and struvite RSS in the urine was investigated in cats in a crossover study design. Increasing potassium up to 1.95% in a dry food formulated for stone dissolution increased urine production by 25% and significantly decreased USG. Several mineral concentrations measured in the urine decreased, including phosphorus, sodium, and sulfate. Urine oxalate concentration was significantly reduced, similar to what was previously reported [19]. Potassium chloride supplementation significantly reduced CaOx RSS. Struvite RSS tended to decrease (*p* = 0.07). These results confirmed that adding potassium to the food could be beneficial for increasing urine production and decreasing CaOx RSS.

The mean age of cats in all 3 studies was ≥11.9 years. There were no adverse health effects noted clinically in these geriatric cats. Blood chemistry results also showed minimal changes. Cholesterol decreased in study 2 in cats receiving 1.81% potassium, as fed, and BUN and glucose decreased in cats receiving 1.95% potassium, as fed, in study 3. Longer duration studies would help inform long-term safety for healthy geriatric cats and cats with renal disease, and are worth conducting, as geriatric and renal disease cats would benefit most from KCl supplementation of foods, as they are at greater risk of CaOx urolithiasis.

More studies have been conducted to assess adding sodium chloride to pet foods. Increased dietary NaCl concentrations increase water intake and urine volume. In one study, diets with differing NaCl content (base diet had sodium 0.3%; NaCl supplemented diets had sodium of 0.7, 1.0, and 1.3%, as fed) were fed to both dogs and cats, and showed a significant increase in water intake and urine volume when NaCl was added to the food [14]. In dogs, increasing NaCl from 0.24% sodium dry matter (DM) to 1.20% and feeding for 6 weeks increased urine volume and urine calcium excretion, although there was no change in urine calcium concentration [21]. Feeding increased dietary sodium (0.2 or 0.3 g sodium/100 kcal food) to two breeds of dogs led to increased production of urine and decreased CaOx RSS [22].

However, in humans, high NaCl intake is associated with increased urine calcium excretion, so it is recommended that NaCl be restricted to prevent CaOx urolith formation. The same is true for cats, in that cats who received varying sodium (0.38 to 1.43% sodium DM) and chloride (0.56 to 2.52% chloride DM) foods produced more urine, but renal calcium excretion also increased, even though urine calcium concentration was unchanged [23]. In addition, oxalate concentrations decreased, and CaOx RSS was unaffected [23]. In study 2, we also saw increased urine calcium excretion, but no change in urine calcium concentration after consuming 1.81% potassium, as fed, for two weeks. In study 3, we observed reduced CaOx RSS after consuming 1.95% potassium, as fed, for two weeks; urine Ca excretion was not measured. In a review of human literature up through 2014 for dietary treatment of risk factors for all types of urolithiasis [24], it was concluded that a mainstay of management is a forced increase in fluid intake, moderate dietary salt restriction to limit urine calcium excretion, and no dietary calcium restriction for adults or children. Replacing dietary salt with potassium was recommended for children.

Studies in dogs and cats have been conducted to assess adding the salt-substitute KCl to foods. In one study, the addition of potassium as KCl (0.44 g/MJ vs 1.03 g/MJ) for 10 days showed water intake and urine volume increased, USG decreased, and CaOx RSS decreased with increased dietary KCl [20]. These findings are similar to ours.

Limitations of these studies include the fact that potassium chloride was used as the potassium supplement in all three trials. Both potassium and chloride are strong ions, and the influence of chloride ions cannot be excluded. Additional studies are needed to determine if a similar response is found if potassium is the only strong ion fed. Another limitation is that the trial durations were relatively short (two to three weeks). Although study durations were long enough to detect differences, longer duration studies are needed to see if differences decline over time. We also did not analyze molecular mechanisms involving renal electrolyte reabsorption or calcium oxalate crystal inhibition in this study. Although we studied different KCl supplementation concentrations, we did not compare potassium sources.

## 5. Conclusions

In summary, these three studies indicate that up to 1.95% potassium in dry food fed to cats is helpful for increasing water intake and urine production, for decreasing USG and concentrations of urine minerals, and for reducing CaOx RSS. Based on physical examination and blood chemistry results, no adverse effects were observed. It would be reasonable to speculate that these changes would result in less stone formation in cats.

## Figures and Tables

**Table 1:** Effect of dietary potassium supplementation on body weight, food intake, feces and urine production, and potassium and chloride excretions in adult cats.

	Control Food	KCl Supplemented Food	*p*-Value
**Potassium content of food, as fed (%)**	1.08	1.95	
**Measures (means ± SE)**			
Body weight on day 21, kg	4.57 ± 0.400	5.02 ± 0.390	0.443
Food intake, g/d	49.0 ± 3.02	56.6 ± 1.80	0.058
Feces production, g/d	10.6 ± 1.20	11.6 ± 1.13	0.577
Urine production, g/d	48 ± 8.2	82 ± 11.5	0.037
Potassium intake, g/d	0.53 ± 0.033	1.10 ± 0.035	<0.001
Fecal potassium, g/d	0.01 ± 0.002	0.01 ± 0.002	0.609
Urine potassium, g/d	0.38 ± 0.057	0.65 ± 0.103	0.049
Chloride intake, g/d	0.49 ± 0.030	1.03 ± 0.033	<0.001
Fecal chloride, g/d	0.01 ± 0.001	0.01 ± 0.002	0.193
Urine chloride, g/d	0.42 ± 0.074	0.87 ± 0.111	0.007
Fecal score	4.0 ± 0.14	4.1 ± 0.09	0.881

**Table 2:** Effect of dietary potassium supplementation on water intake, food intake, urine production, urine mineral concentrations, and daily urine mineral excretions in adult cats.

	KCl Supplemented Food				Adjusted *p*-Values		
	A	B	C	SEM	A vs. B	A vs. C	B vs. C
**Potassium content of food, as fed (%)**	0.84	1.35	1.81				
**Measures (means ± SE)**							
Water intake, g/d	79.3	85.1	100.0	10.2	0.210	<0.001	<0.001
Food intake, g/d	43.14	42.42	43.01	1.99	0.765	0.988	0.845
Urine production, g/d	37.15	45.43	53.14	3.70	0.007	<0.001	0.011
Urine specific gravity	1.057	1.050	1.044	0.002	<0.001	<0.001	<0.001
Urine pH	6.62	6.77	6.98	0.15	0.357	0.011	0.153
Urine mineral concentrations							
Phosphorus, mg/kg	2148	1754	1409	70	<0.001	<0.001	<0.001
Magnesium, mg/kg	82.1	63.0	49.7	10.1	0.388	0.086	0.623
Calcium, mg/kg	53.53	54.33	54.99	4.98	0.986	0.953	0.990
Sodium, mg/kg	3552	3059	2361	142	0.017	<0.001	0.002
Potassium, mg/kg	9686	13,211	14,611	544	<0.001	<0.001	0.027
Chloride, mg/kg	10,768	13,079	13,770	566	0.003	<0.001	0.474
Urine mineral excretion, mg/d							
Phosphorus, mg/d	79.22	78.15	73.63	4.64	0.952	0.291	0.436
Magnesium, mg/d	2.79	2.72	2.44	0.41	0.990	0.815	0.883
Calcium, mg/d	2.01	2.47	2.86	0.30	0.149	0.006	0.248
Sodium, mg/d	130.69	135.80	122.63	7.18	0.780	0.549	0.222
Potassium, mg/d	356	589	757	29	<0.001	<0.001	<0.001
Chloride, mg/d	390	582	710	29	<0.001	<0.001	0.003
Body weight (BW) change, ^1^ %	−3.40	−3.10	−3.34	0.76	0.482	0.487	1.000

^1^ (BW at end of treatment period) − (BW at beginning of treatment period) × 100/(BW at beginning of treatment period).

**Table 3:** Effect of dietary potassium supplementation on serum chemistries in adult cats in study 2.

	KCl Supplemented Food				Adjusted *p*-Values		
	A	B	C	SEM	A vs. B	A vs. C	B vs. C
**Potassium content of food, as fed (%)**	0.84	1.35	1.81				
**Measures (means ± SE)**							
Albumin, g/dL	3.267	3.256	3.222	0.088	0.973	0.646	0.779
ALP, U/L	25.78	26.44	26.44	4.63	0.877	0.877	1.000
ALT, U/L	54.33	60.67	64.11	5.38	0.262	0.058	0.657
BUN, mg/dL	19.64	16.67	18.04	1.60	0.294	0.688	0.756
Cholesterol, mg/dL	192.9	186.0	172.2	13.4	0.393	0.003	0.043
Creatinine, mg/dL	1.167	1.189	1.156	0.066	0.637	0.890	0.375
Glucose, mg/dL	74.56	74.78	75.89	2.04	0.994	0.793	0.852
Total bilirubin, mg/dL	0.144	0.122	0.167	0.026	0.713	0.713	0.282
Total protein, g/dL	6.92	6.90	6.90	0.18	0.978	0.978	1.000
Triglycerides, mg/dL	26.00	29.56	29.44	4.32	0.453	0.475	0.999
Calcium, mg/dL	10.13	9.88	9.87	0.15	0.199	0.174	0.997
Phosphorus, mg/dL	4.09	3.95	3.95	0.23	0.480	0.513	0.998
Chloride, mmol/L	124.22	124.11	123.89	0.47	0.954	0.663	0.830
Magnesium, meq/L	2.54	2.71	2.42	0.14	0.659	0.794	0.304
Potassium, mmol/L	5.13	4.87	5.14	0.11	0.245	0.997	0.220
Sodium, mmol/L	157.67	157.44	157.44	0.50	0.860	0.860	1.000

**Table 4:** Effect of dietary potassium supplementation on body weight, food intake, urine production, and urine mineral concentrations in adult cats.

Food	Control	KCl Supplemented	SEM	*p*-Value
**Potassium content of food, as fed (%)**	1.08	1.95		
**Measures (means ± SE)**				
Body weight change, %	0.57	−0.52	0.71	0.101
Food intake, g/d	41.31	40.41	1.50	0.232
Urine production, g/d	34.20	42.85	3.46	0.004
Urine specific gravity	1.0513	1.0416	0.0026	<0.001
Urine pH	6.956	6.999	0.085	0.575
Urine mineral concentrations				
Phosphorus, mg/kg	1982	1324	93	<0.001
Magnesium, mg/kg	57.40	53.01	5.10	0.523
Calcium, mg/kg	53.93	50.83	7.82	0.513
Sodium, mg/kg	3159	2508	162	0.001
Potassium, mg/kg	10,735	14,686	699	<0.001
Chloride, mg/kg	9400	12,010	630	<0.001
Urine Oxalate, µM	856	711	36	0.003
Urine Citrate, mM	2.34	2.52	0.47	0.633
Urine Sulfate, mM	46.34	30.87	2.67	<0.001
Urine RSS CaOx, no Cr covariate	3.48	2.96	0.41	0.019
Urine RSS CaOx, with Cr covariate	3.54	2.90	0.37	0.007
RSS Struvite, no Cr covariate	4.39	3.45	0.78	0.103
RSS Struvite, with Cr covariate	4.48	3.37	0.76	0.071
Fecal score	4.40	4.39	0.15	0.890

**Table 5:** Effect of dietary potassium supplementation on serum chemistries in adult cats in study 3.

Food	Control	KCl Supplemented	SEM	*p*-Value
**Potassium content of food, as fed (%)**	1.08	1.95		
**Measures (means ± SE)**				
Albumin:globulin ratio	0.760	0.780	0.038	0.347
Albumin, g/dL	2.930	2.900	0.082	0.511
ALP, U/L	22.80	23.10	2.26	0.613
ALT, U/L	59.2	54.3	5.6	0.232
BUN, mg/dL	19.58	18.49	0.90	0.026
Cholesterol, mg/dL	168.8	169.9	9.9	0.767
Creatinine, mg/dL	1.320	1.280	0.060	0.111
Glucose, mg/dL	75.00	71.80	1.01	0.042
Total bilirubin, mg/dL	0.130	0.120	0.014	0.545
Total protein, g/dL	6.85	6.74	0.17	0.406
Triglycerides, mg/dL	27.1	29.9	3.4	0.523
Calcium, mg/dL	9.65	9.70	0.20	0.786
Phosphorus, mg/dL	4.44	4.36	0.21	0.363
Chloride, mmol/L	122.10	121.60	0.41	0.392
Magnesium, meq/L	1.640	1.590	0.035	0.066
Potassium, mmol/L	4.89	4.92	0.12	0.847
Sodium, mmol/L	159.00	158.30	0.48	0.174

## Data Availability

All data are included in the manuscript and are freely available upon request.

## Abbreviations

The following abbreviations are used in this manuscript:

AAFCO	Association of American Feed Control Officials
BW	body weight
CaOx	calcium oxalate
CBC	complete blood count
DM	dry matter
DSH	domestic short-hair
KCl	potassium chloride
KHCO_3_	potassium bicarbonate
NaCl	sodium chloride
RSS	relative supersaturation
SEM	standard error of the mean
USG	urine specific gravity
